# From Waste to Wires: PBAT/Lignin Biocomposites Functionalized by a CO_2_ Laser for Transient Electronics

**DOI:** 10.3390/polym17233144

**Published:** 2025-11-26

**Authors:** Antonella Moramarco, Elio Sarotto, Itziar Otaegi, Nora Aranburu, Federico Cesano, Valentina Brunella, Marco Zanetti, Pierangiola Bracco

**Affiliations:** 1 Department of Chemistry, University of Torino, via Pietro Giuria 7, 10125 Turin, Italyfederico.cesano@unito.it (F.C.); marco.zanetti@unito.it (M.Z.); 2 POLYMAT and Department of Advanced Polymers and Materials: Physics, Chemistry and Technology, Faculty of Chemistry, University of the Basque Country UPV/EHU, Paseo Manuel de Lardizabal 3, 20018 Donostia-San Sebastián, Spainnora.aramburu@ehu.eus (N.A.); 3 SUSPLAS@UniTo, Sustainable Plastic Scientific Hub, University of Torino, Via Pietro Giuria 7, 10125 Turin, Italy; 4 NIS Interdepartmental Centre, University of Torino, 10125 Turin, Italy

**Keywords:** PBAT, lignin, glass fibers, transient electronics, laser functionalization, biodegradable polyester, polymer composites

## Abstract

Polybutylene adipate terephthalate (PBAT), a flexible biodegradable polyester, has gained widespread use in packaging applications due to its ability to degrade under controlled conditions, producing non-toxic substances. While this property makes PBAT particularly attractive for the development of transient electronic devices, this potential application remains unexplored. To address this research gap, we developed PBAT-based composites and modified their electrical properties through CO_2_ laser functionalization. Although laser treatment of neat PBAT primarily resulted in material ablation, the incorporation of lignin and silica-based fillers enabled the formation of electrically conductive pathways. Among the various fillers tested, dealkaline lignin (DEALK) and glass fibers (GFs) provided the optimal combination of electrical conductivity, mechanical properties, and processability. Characterization techniques (electrical measurements, optical microscopy, SEM, EDX, and TGA) highlighted that by optimizing laser treatment and the filler concentration, it is possible to produce conductive tracks with remarkably low sheet resistance. Hybrid composites containing 10–15 wt% of GF and 20–25 wt% of lignin demonstrated the best electrical performance with values as low as 3.5 Ω/sq, which were further reduced to 1.72 Ω/sq after laser process optimization. These findings establish PBAT composites as promising candidates for sustainable transient electronics.

## 1. Introduction

The increasing demand for electronic devices and the programmed obsolescence of these products annually generate large amounts of electronic waste (e-waste). According to the United Nations Institute for Training and Research, 62 million tons of e-waste were produced worldwide in 2022, and only 22.3% of it was properly collected and recycled [1]. The difficulties associated with recycling (complexity of devices, presence of hazardous components, cost) lead to the disposal of large amounts of electronic waste in landfills, with tremendous consequences for the environment and human health [2]. In recent years, a new class of electronics has been developed that could partially solve the problem of e-waste pollution and management. This technology, known as *transient electronics*, is thought to exist for a limited time and disappear completely or partially at controlled rates [3,4,5,6]. Disintegration of these devices is regulated through chemical, electrochemical, and mechanical processes, resulting in the formation of safe byproducts [4]. Transient electronics eliminate the risks associated with conventional electronics, which are responsible for the release of hazardous chemicals and toxic metals into the environment [3,7]. The innovative concept of transient electronics paves the way for new opportunities, especially for the production of temporary medical implants that can reduce the need for a second surgery and decrease the infection risks associated with device retrieval. Although this technology is mainly intended for biomedical applications, transient devices can also be employed in the defense sector to fabricate secure hardware systems and for the production of environmentally friendly devices [5,8]. Electronic devices are multicomponent objects in which each layer provides a distinct function. The substrate provides mechanical support and insulates the electronics; it is thicker and larger than the other layers and generates the largest amounts of e-waste. Dielectric, semiconductor, and conductor materials are used to manage the electric signal, whereas the encapsulation layer has a protective function [3,7]. Some metals and inorganic materials, the key components in conventional electronics, are promising candidates for transient devices. The dissolution kinetics, biodegradability, and biocompatibility of these materials are being investigated for their applicability in eco-friendly and biomedical devices [4,5]. Natural and synthetic polymers offer several advantages over inorganic materials, as they are soft, flexible, and stretchable. In addition, their electrical and mechanical properties can be easily tuned through chemical structural modifications, enabling their applicability as substrates, dielectrics, semiconductors, and encapsulating materials [3,6]. Due to their unique properties, polymers are used for the production of flexible and biodegradable devices, which find applications in several fields, such as the biomedical sector [9,10,11].

Although transient electronics show considerable advantages, the production of these complex devices requires several manufacturing steps. A novel process has been developed that enables the fabrication of electronic devices starting from a single composite material that contains either a conductive filler or a graphene-like structure precursor. This technology uses a laser to scribe conductive tracks within an insulating matrix [12]. The laser modifies the local electrical properties of composites by promoting two distinct phenomena: it ablates the polymeric matrix, resulting in a local increase in the filler concentration [13], and it partially carbonizes char-forming polymers and fillers, promoting the formation of graphene-like and other conductive carbonaceous structures [14,15]. When the concentration of conductive fillers (such as carbon nanotubes, graphene, and carbon black) exceeds the percolation threshold after laser treatment, the material’s electrical behavior is modified, transitioning from insulating to conductive [16]. In addition to these functional modifications, laser technology offers numerous advantages, including the possibility of developing flexible devices [17], reducing manufacturing steps, and making electronic devices using a single, easily recyclable material [18].

Polybutylene adipate terephthalate (PBAT) is an aliphatic–aromatic biodegradable copolyester widely used in the packaging sector [19,20]. This material exhibits great flexibility and biodegradability and has recently been studied for the development of flexible transient electronic devices [21,22]. However, the poor thermomechanical and tensile properties of PBAT limit its use. To overcome these limitations and broaden its applicability, PBAT-based blends and composites have been developed [19,20].

Lignin is the second most abundant natural polymer and is one of the main byproducts of the paper manufacturing and biorefinery industries, which produce over 50 million tons of lignin every year [23]. Lignin is a hydrophobic aromatic polymer and consists of three structural units randomly connected by C-C and C-O bonds, forming a three-dimensional network [24,25]. This complex structure contains several functional groups, such as phenolic, hydroxyl, carboxyl, carbonyl, and methoxy groups, that endow lignin with unique features, e.g., fluorescence and UV-blocking ability [26]. The type of original biomass and the isolation process affect its structure and properties [27,28,29]. For example, Wang et al. [30] observed variations in the pyrolysis behavior of lignin extracted from pine wood through different chemical treatments. More in detail, the lignin isolated using alkaline solutions exhibited higher char-forming ability, thanks to the catalytic effect of sodium cations [31], and lower thermal stability than the lignin isolated with organic solvents.

Several studies demonstrate that the incorporation of lignin into polymeric matrices enhances the performance of the composites by imparting antimicrobial activity, antioxidant capacity, and light stability [23]. Nevertheless, the poor compatibility of lignin with many polymers, e.g., PBAT, leads to self-agglomeration of filler particles and progressive worsening of mechanical performance as the lignin content increases [23,32]. The strategies developed to enhance the mechanical properties of PBAT/lignin composites involve the modification of lignin and/or the addition of specific modifiers [23,32,33,34,35,36,37,38]. Gu et al. [32] observed an increase in the tensile properties of PBAT/lignin composites with the incorporation of hydrophobic silica nanoparticles, while hydrophilic silica nanoparticles did not improve the material performance.

The outstanding performance of PBAT/lignin composites, such as biodegradability, UV protection, and barrier properties [23,32,34,35,36,37,38,39,40], makes them excellent candidates for the food packaging sector [37,40]. Furthermore, the presence of extended aromatic and conjugated carbon structures makes lignin an excellent candidate to obtain conductive graphene-like structures through laser treatment [41,42]. Although these biodegradable materials are suitable for developing transient electronics, to the best of our knowledge, their application in this sector has not yet been explored. To address this gap, we prepared composites containing PBAT, lignin, and silica and modified their electrical properties through CO2 infrared laser treatment. This study was designed to systematically assess the individual contributions of two different types of lignin (alkaline and dealkaline ones) and various silica-based fillers (hydrophobic and hydrophilic nanoparticles and glass fibers). The rationale behind this selection is based on the observation that laser irradiation under an inert atmosphere induces lignin pyrolysis and promotes the formation of carbonaceous conductive structures, due to the reduction of oxygenated functional groups (e.g., carbonyl, carboxyl, and hydroxyl groups) and the consequent formation of conjugate double bonds [43,44,45,46]. Concurrently, silica-based fillers were evaluated for their capacity to enhance the tensile properties of PBAT and the mechanical stability of the conductive tracks produced by the laser treatment. In the first part of this study, we investigated the influence of the different fillers on the performance of PBAT by preparing samples in which the type of filler was varied while keeping the filler concentrations constant. Fillers that improved the tensile and electrical properties of the polymer were selected and used in the second part of the study to prepare composites with varying filler concentrations. This part of the work aimed to correlate the electrical performance of the composites with the filler content.

## 2. Materials and Methods

### 2.1. Materials

PBAT (TECHNIPOL Bio 1160) was purchased from SIPOL (Mortara, PV, Italy), the two types of lignin (alkaline and dealkaline) from Tokyo Chemical Industry (Tokyo, Japan), the hydrophilic (CAB-O-SIL LM-150 fumed silica) and hydrophobic silica (CAB-O-SIL TS-530 fumed silica) from CABOT (Boston, MA, USA), and the glass fibers from PROCHIMA (Terzo, AL, Italy). All the materials were oven-dried at 60 °C overnight before use. The properties of the fillers used are summarized in Table 1 and Table 2.

### 2.2. Methods

**Figure 1:**
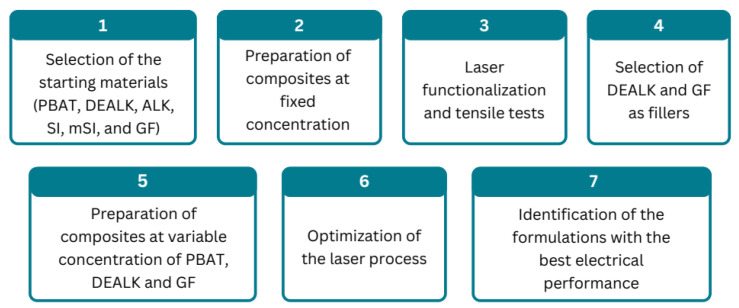
Graphical representation of the methodology used in this study.

Composites containing different amounts and types of fillers were prepared with a Collin twin-screw extruder-kneader ZK25 (Maitenbeth, Bavaria, Germany), operating at 140 °C and 200 rpm as screw rotation speed. The melted compounds were chilled in a water bath and pelletized. The pellets were oven-dried at 60 °C overnight, then they were processed in a Battenfeld PLUS 350/75 injection molding machine (Kottingbrunn, Austria) at 140 °C to obtain bars 6.5 cm long, 1.3 cm wide, and 6 mm thick. A complete list of the composites prepared in this study, along with their theoretical compositions, is provided in Table S1, while a flowchart illustrating the methodology used is shown in Figure 1. The samples are labeled with the type and the theoretical amounts of lignin and silica within the composite. The dealkaline and alkaline lignin materials are denoted as DEALK and ALK, respectively, while hydrophilic silica, hydrophobic silica, and glass fibers are represented as SI, mSI, and GF, respectively. For example, the code 30DEALK_5GF refers to a sample containing 30 wt% dealkaline lignin, 5 wt% glass fibers, and 65 wt% PBAT.

Tensile tests were performed on selected samples. The tensile specimens (ASTM D-638 type IV) were prepared at 140 °C using a Battenfeld PLUS 350/75 injection molding machine (Kottingbrunn, Austria). The mechanical tests were performed with an Instron 5569 tensile tester (Norwood, MA, USA), following ASTM D-638 and using a crosshead speed of 10 mm/min. The modulus, yield stress, stress at break, and nominal strain at break were calculated from stress–strain curves. At least five specimens were tested for each composite.

Thermogravimetric analysis (TGA) was performed on pristine materials and their composites, using a TA Instruments Q500 thermogravimetric analyzer (New Castle, DE, USA) and a heating rate of 20 °C/min. The sample (20–30 mg) was heated from room temperature to 700 °C under nitrogen flux and from 700 °C to 800 °C under air flux. When analyzing pristine lignin, an isotherm of 10 min was carried out in air at 800 °C after the heating ramp. Since the presence of moisture significantly affects residue quantification, the weight percentages were calculated using the following equation:

$$Weight\;\%\left(T\right)=\frac{Weight\;(T)}{Weight\;(150\;{}^{\circ}C)}*100$$

where Weight (T) is the mass of the sample at a given temperature, and Weight (150 °C) is the mass of the sample at 150 °C, corresponding to the dry mass.

A CO_2_ laser engraving machine (4040, Hauser Laser Equipment) was used to treat the composites to obtain conductive tracks. The used equipment operated at a 10.6 μm wavelength in continuous wave (CW) mode with a maximum power of 50 W. Laser treatments were conducted with a 5 L/min N_2_ gas flow condition, with the nozzle placed at the laser head focusing lens to prevent sample burning and damage to the instrument. The N_2_ flux displaces ambient air from the interaction zone and limits oxidative degradation of the precursor, while assisting heat removal and ejection of pyrolysis products. The setup does not constitute a sealed inert chamber, so small amounts of air are still present. A parametric study was conducted to evaluate the effects of key processing parameters on the functionalization process, including laser speed (1–4 mm/s), power (from 8% to 12%, more specifically from 2.0 W to 4.6 W), focal point position (0–5 mm), number of passes (one or two), and pretreatment step. No in situ temperature monitoring was performed during CO_2_ laser treatments.

The terminal parts of the obtained conductive tracks were painted using Ag paste to ensure a good electrical connection with the Cu leads linked to the multimeter. DC electrical measurements were performed using the two-probe method, connected to a Keithley 2420 source meter (Keithley Instruments, Solon, OH, USA). The sheet resistance was calculated using the following equation:

$$R_{S}=\frac{R*w}{l}$$

where RS is the sheet resistance, R is the measured resistance in Ohms (Ω), w is the width, and l is the length of the track.

The assessment of filler dispersion was based on qualitative but systematic scanning electron microscopy (SEM) observations on multiple regions and magnifications, using a Tescan VEGA3 microscope (Brno, Czech Republic). The pictures were recorded at 5 keV and 30 pA using secondary electrons. Before the analysis, the samples were cryogenically fractured in liquid nitrogen and then coated with a layer of gold using a VacCoat DSR1 sputter coater (London, UK) to prevent charging and sample damage.

The morphology of the conductive tracks was investigated with electron and optical microscopies. A Leica EZ4 D (Wetzlar, Germany) optical microscope was used to evaluate the profile and length of the tracks. SEM analysis was performed using an acceleration voltage of 10 keV and a beam current of 30 pA. Since the samples behaved as electrical conductors, they were not gold sputtered before SEM analysis.

Energy-dispersive X-ray (EDX) analysis was performed with a Tescan VEGA3 microscope coupled to an Ultim Max 40 detector by Oxford Instruments (Abingdon, UK). The chemical composition is expressed as weight percent (wt%).

## 3. Results and Discussion

### 3.1. Preliminary Tests

Lignin is a natural and low-cost filler that can produce graphitic structures when subjected to thermal treatments or laser writing under controlled conditions [15]. Lignin is thermally stable up to 200 °C (TGA shown in Figure S1) and can be processed with PBAT by melt mixing techniques without undergoing thermomechanical degradation. The preliminary investigations served a dual purpose: to identify the optimal laser parameter sets and to determine which filler types most effectively enhance electrical conductivity while maintaining desirable mechanical properties and processability. In this section, we show the results of samples containing different types of lignin and silica, while keeping the concentration constant. More precisely, the results of the composites containing 5 wt% silica (SI, mSI, and GF) and/or 30 wt% lignin (ALK, DEALK) are reported.

#### 3.1.1. Mechanical Properties

**Figure 2:**
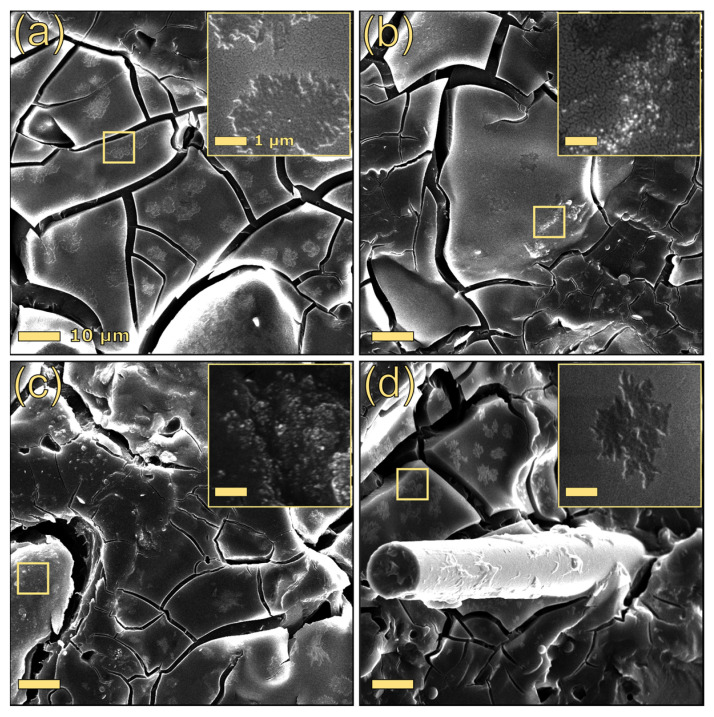
SEM images of 30ALK (**a**), 30ALK_5SI (**b**), 30ALK_5mSI (**c**), and 30ALK_5GF (**d**). The scale bar of the primary images is 10 µm, whereas the bar of the insets is 1 µm.

**Figure 3:**
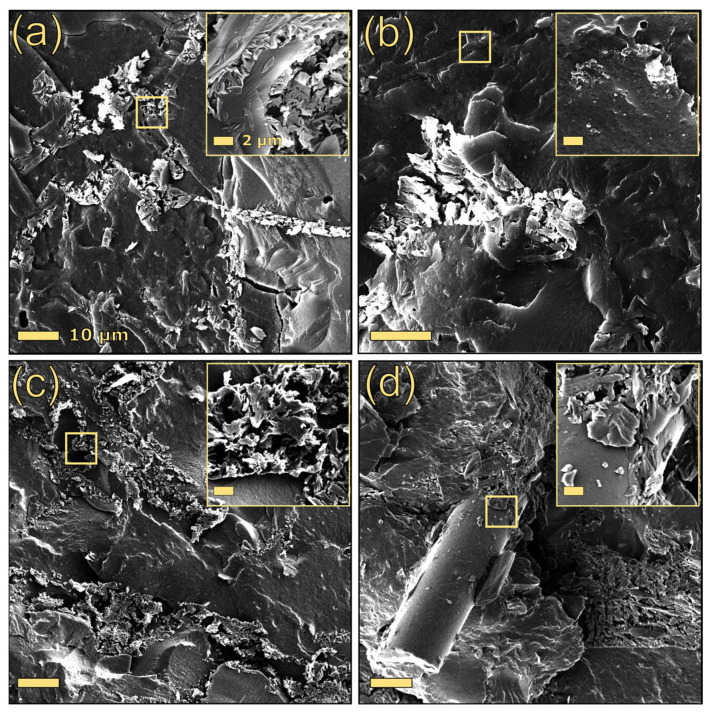
SEM images of 30DEALK (**a**), 30DEALK_5SI (**b**), 30DEALK_5mSI (**c**), and 30DEALK_5GF (**d**). The scale bar of the primary images is 10 µm, whereas the bar of the insets is 2 µm.

The stress–strain curves of PBAT-based composites are reported in the Supplementary Material (Figure S3). As can be seen from Table 3, which summarizes the most interesting tensile parameters, the addition of lignin to PBAT substantially increased its Young’s modulus, regardless of the type of lignin, indicating an improvement in the material’s stiffness. However, the incorporation of lignin resulted in a reduction in yield stress, stress at break, and strain at break. Microstructural analysis of these composites revealed that alkaline lignin formed irregularly shaped particles, ranging in length from 2 to 7 µm, within the polymer matrix (Figure 2a). Conversely, the dealkaline lignin particles were not clearly distinguishable from the matrix (Figure 3a). The reduced propensity for dealkaline lignin to form large aggregates within PBAT may partially justify the superior tensile properties of 30DEALK compared to 30ALK.

As reported in the literature, the presence of irregularly shaped fillers and their poor interfacial adhesion with the polymeric matrix cause a reduction in the tensile strength of the composite material [47]. With the aim of improving the tensile properties of PBAT/lignin composites, three silica-based fillers, differing in dimensionality, shape, and surface chemistry, were incorporated. While glass fibers (GFs) and unmodified silica (SI) did not enhance the tensile properties of alkaline lignin formulations, hydrophobic silica (mSI) significantly increased their yield strength. Given the comparable dispersion states of both nano silicas (Figure 2), the improved mechanical performance of 30ALK_5mSI may be ascribed to the superior compatibility between silica, PBAT, and alkaline lignin. Similar results were observed by Gu et al. [32] for PBAT-based composites containing a different type of lignin (sodium lignosulfonate).

In contrast, the inclusion of silica-based fillers in the composite PBAT/dealkaline lignin led to a deterioration in its mechanical properties, regardless of the type of silica added. As silica incorporation did not significantly affect lignin particle dispersion (Figure 3), the observed reduction in tensile performance may be ascribed to poor interfacial compatibility between the constituent materials. Among the formulations containing both dealkaline lignin and silica-based fillers, 30DEALK_5GF demonstrated a higher Young’s modulus than their counterparts with silica nanoparticles.

Overall, the addition of lignin and silica to PBAT enhanced its tensile modulus, thereby extending the potential applicability of this biopolymer to fields requiring higher stiffness.

#### 3.1.2. Electrical Properties

**Figure 4:**
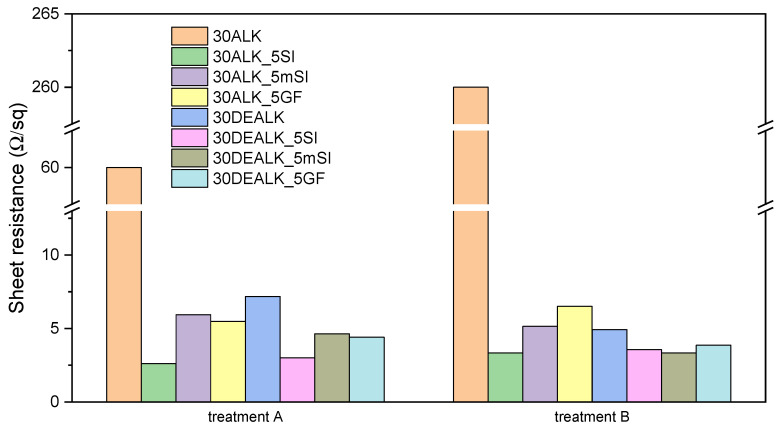
Electrical sheet resistance values obtained by performing laser treatment of various PBAT-based composites filled with different percentages of lignin (alkaline and dealkaline) and silica-based fillers (hydrophilic nanoparticles, hydrophobic nanoparticles, and glass fibers). The resistance values were measured on 20 mm long tracks produced under a nitrogen atmosphere and subsequently connected with silver paste.

The composites were subsequently functionalized with a CO_2_ laser by systematically varying the speed, power, and focal length. N_2_ flow rate and gas purity were kept constant and were not treated as independent process parameters. Under these conditions, no signs of significant oxidation (such as burning or complete ablation of the carbonized region) were observed, and both track morphology and sheet resistance were reproducible across samples. Initial experiments using high laser powers resulted in significant substrate degradation and poor mechanical stability. In contrast, high speeds produced narrow, deep grooves that lacked electrical conductivity. Due to their poor performance, the results of the treatments using high speeds and powers will not be further discussed. Two laser treatments (A and B) were selected for detailed investigation as they yielded promising results. These two parameter sets, characterized by low laser writing speed (1 mm/s) and low laser power (8%), differ solely in the focal point position. Treatment A utilized a surface-focused beam, while treatment B employed a 5 mm overfocus. Both configurations yielded optimal results, generating conductive tracks with low electrical resistance (see Figure 4) and enhanced stability under mechanical stress.

**Figure 5:**
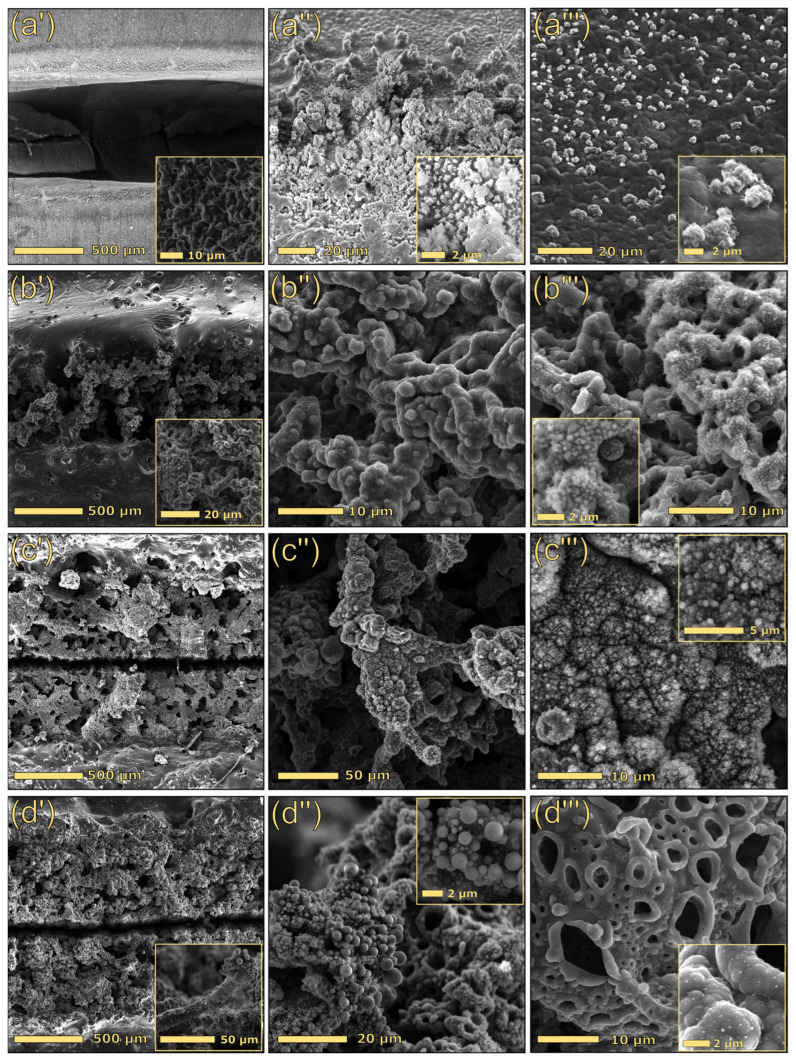
Top-view images under different magnifications of the laser-scribed tracks for neat PBAT (**a′**–**a‴**), 30DEALK (**b′**–**b‴**), 30DEALK_5mSi (**c′**–**c‴**), 30DEALK_5GF (**d′**–**d‴**). The tracks were obtained using treatment A.

The laser treatments performed on pristine PBAT did not lead to conductive tracks. Indeed, even though PBAT is a char-forming polymer, the laser primarily promoted the ablation of the material, and the formation of carbonaceous tracks was not macroscopically observed (Figure S4). We hypothesize that this phenomenon may be attributed to two main factors: first, PBAT produces only a small amount of char during pyrolysis (3.7 wt%); second, the absence of a high-temperature stable filler (e.g., silica), which could stabilize the small amount of carbonaceous residue formed during the laser treatment, leads to a predominant ablation mechanism. Morphological analysis confirmed this hypothesis. Notably, the primary interaction mechanism between the laser beam and neat PBAT (Figure 5(a′)) led to polymer ablation, as evidenced by an extensively excavated region approximately 700 µm wide. Higher magnification examination (Figure 5(a‴)) revealed the presence of a small quantity of sputtered material along the external path edges (Figure 5(a″)), probably consisting of partially carbonized polymer.

Considering the electrical results illustrated in Figure 4, the two optimized laser treatments produced analogous trends. The composite with 30 wt% alkaline lignin showed the worst electrical performance: the electrical sheet resistance is 8-fold and 52-fold the corresponding composite containing dealkaline lignin when treatment A and B were applied, respectively. The inferior electrical properties of the 30ALK formulation may be correlated with inadequate filler dispersion. Notably, while the incorporation of silica-based fillers induced no significant microstructural modifications in the alkaline lignin composites, it resulted in a pronounced decrease in electrical resistance. Specifically, the sample with 30 wt% alkaline lignin and 5 wt% hydrophilic silica resulted in the best electrical performance, showing a sheet resistance of 2.61 Ω/sq after the laser treatment A. Similarly, the laser process with a 5 mm overfocus condition resulted in a substantial reduction in the electrical resistance of the sample, leading to a sheet resistance that was 78 times lower than that of the composite without silica (30ALK).

Similarly, adding silica-based fillers to 30DEALK enhanced its electrical performance. Although the incorporation of silica-based fillers resulted in a less dramatic, albeit still significant, decrease in electrical resistance, these composites exhibited superior track stability and morphology compared to 30DEALK.

However, it is paramount to highlight that when the melted composites were quenched in the water bath, lignin leaching occurred exclusively in formulations containing the alkaline type. This phenomenon indicated that alkaline lignin-based composites may have restricted practical applicability because of their poor stability in water.

Considering the tensile and electrical results, in addition to the enhanced processability of dealkaline lignin and glass fibers, this combination was selected for further observation and subsequently employed in the second part of this study to fabricate a series of composites for further evaluation.

Figure 5 displays the morphology of laser-scribed tracks, produced using identical scribing parameters (treatment A) for different material compositions. Notably, the incorporation of lignin, a char-forming biopolymer, into PBAT was pivotal for the formation of conductive tracks under laser irradiation. The generation of conductive structures is attributed to lignin carbonization, the concurrent reduction of all oxygen-containing functional groups, and the formation of conjugated double bonds, in line with previous studies on lignin-derived laser-induced graphene, where Raman, XRD, and electron microscopy confirmed the formation of graphene-/graphite-like domains after CO_2_ laser writing [45]. Although direct structural analysis of the graphitic domains in our tracks was not carried out here, the observed branched carbonaceous morphology and the strong dependence of sheet resistance on lignin content and laser parameters are consistent with the formation of such conductive sp^2^–carbon networks [44,48].

Low-magnification images (first column of Figure 5) revealed a significant variation in material response to the laser beam, depending on the composition. Specifically, the track generated on 30DEALK (Figure 5(b′)) exhibited a width of approximately 700 µm and a less defined morphology compared to formulations that incorporated silica-based fillers. Conversely, both 30DEALK_5mSi and 30DEALK_5GF displayed broader tracks, measuring between 1.2 and 1.3 mm in thickness. This difference is attributable to a scattering effect induced by the silica, which promoted a more homogeneous interaction between the laser beam and the material, consequently yielding a larger conductive track. Furthermore, these conductive paths demonstrated a well-defined structure characterized by a central void flanked by densified conductive material. The enhanced morphological stability may be attributed to a physical stabilization effect induced by the incorporation of silica, which facilitated the formation of well-defined edges with reduced defects.

Higher-magnification SEM imaging revealed that the conductive paths exhibited a complex morphology characterized by intricate branching patterns decorated with homogeneously dispersed carbon particles (insets in Figure 5(b‴,c‴)). This distinctive structural feature was consistently observed in all the composites containing lignin. The inclusion of glass fibers resulted in a more complex morphology characterized by the branched structures previously described and spherical particles (Figure 5(d″)). The appearance of these spherical structures suggests that the laser treatment promoted a partial morphological modification of glass fibers. Although the filler predominantly retained its fibrous morphology (see inset in Figure 5(d′)), localized laser-induced melting produced micron-scale spherical particles. EDX analysis (Figure S5) revealed that these particles are principally composed of glass spheres coated with a carbon layer.

### 3.2. Study of Composites Containing PBAT, Dealkaline Lignin, and Glass Fibers

#### 3.2.1. Optimization of the Electrical Properties

**Figure 6:**
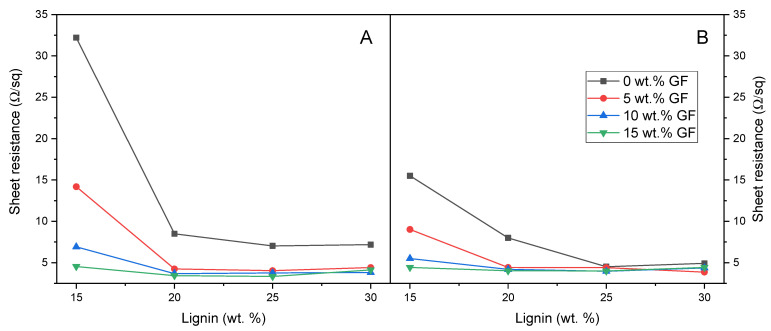
Comparison of the electrical properties in terms of sheet resistance for composites containing varying percentages of dealkaline lignin and GF: (**A**) values obtained using treatment A; (**B**) values obtained using treatment B. All measurements were taken from 20 mm long tracks.

**Figure 7:**
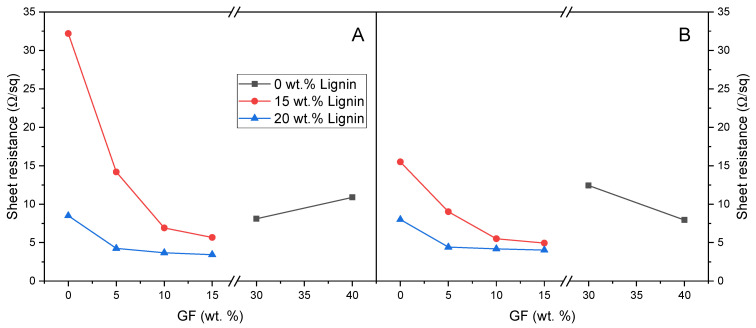
Comparison of the electrical properties in terms of sheet resistance for composites containing varying percentages of GF and dealkaline lignin: (**A**) values obtained using treatment A; (**B**) values obtained using treatment B. All measurements were taken from 20 mm long tracks.

Composites containing variable amounts of dealkaline lignin and glass fibers were subjected to the laser treatments discussed in Section 3.1.2. The obtained electrical properties are illustrated in Figure 6 and Figure 7.

For composites containing PBAT and dealkaline lignin (see the black line in Figure 6), an increase in lignin content, under constant laser treatment conditions, resulted in a reduction in sheet resistance. This decrease was particularly marked in the range between 15 wt% and 20 wt% lignin for the surface-focused treatment (Panel A), where resistance values decreased from 32.2 Ω/sq to 8.5 Ω/sq.

Beyond this range, further increases in lignin content produced relatively stable electrical properties, converging to approximately 7 Ω/sq. In contrast, the overfocus treatment (Panel B) yielded overall lower sheet resistance values, with a gradual decline from 15.5 Ω/sq at 15 wt% lignin load to values below 5 Ω/sq for formulations containing 25 wt% and 30 wt% lignin.

The incorporation of glass fibers within composites containing lignin yielded a substantial enhancement in the electrical conductivity. Specifically, increasing glass fiber content resulted in progressively lower sheet resistance values, reaching as low as 3.44 Ω/sq in the formulation with 20 wt% lignin and 15 wt% glass fibers. It is worth observing that 15DEALK_15GF exhibited significantly lower sheet resistance (4.54 Ω/sq under treatment A) compared to formulations containing either 30 wt% GF (8.11 Ω/sq) or 30 wt% DEALK (7.18 Ω/sq) alone. This marked reduction in electrical resistance suggested a synergistic effect between the two fillers.

It is noteworthy that certain formulations without lignin demonstrated promising electrical properties after laser functionalization (Figure 7). However, in these composites containing only glass fibers as filler, adequate conductivity was only attained with a sufficiently high fiber loading.

**Figure 8:**
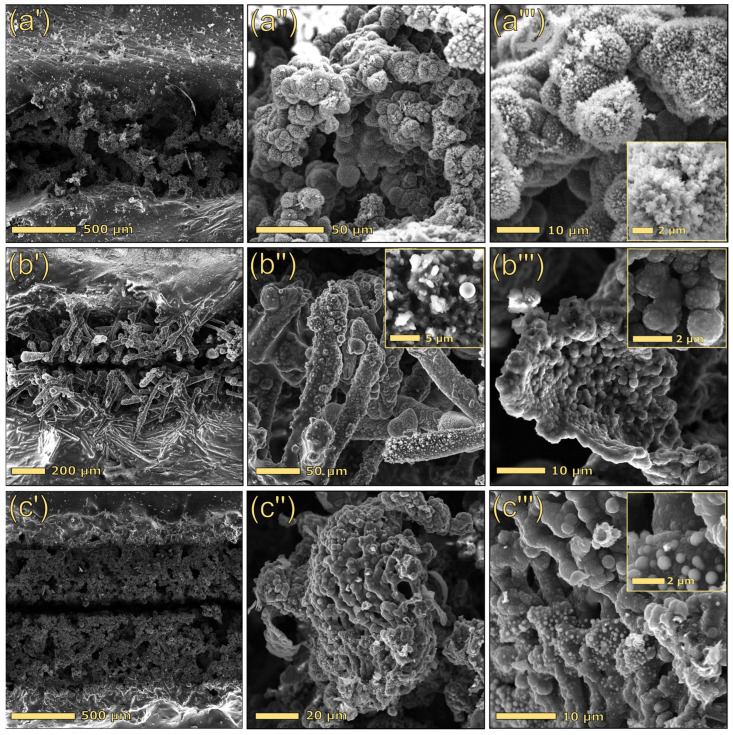
Top-view images at different magnifications of the conductive tracks for 15DEALK (**a′**–**a‴**), 30GF (**b′**–**b‴**), and 25DEALK_15GF (**c′**–**c‴**). The tracks were obtained using treatment A.

For a better interpretation of the electrical results, SEM analysis was performed on selected formulations. Figure 8 compares the morphology of the tracks scribed on three distinct formulations containing solely dealkaline lignin (15DEALK, Figure 8(a′–a‴)), solely glass fibers (30GF, Figure 8(b′–b‴)), and a composite incorporating both lignin and glass fibers (25DEALK_15GF, Figure 8(c′–c‴)), which exhibited optimal electrical properties. All tracks were generated using identical laser parameters (treatment A). A comparison of the laser-scribed track morphologies in composites with varying lignin content (15 wt% vs. 30 wt%) revealed that while the macroscopic profiles of the tracks were similar between 15DEALK (Figure 8(a′)) and 30DEALK (Figure 5(b′)) samples, their microscopic architectures exhibited significant differences. Specifically, when 15DEALK was subjected to the laser process, the laser beam induced carbonization of lignin, producing branched structures decorated with homogeneously dispersed carbon particles (Figure 8(a″)). While 15DEALK exhibited a poorly interconnected network, 30DEALK demonstrated more entangled conductive structures that facilitated charge carrier mobility, yielding lower sheet resistance values (see Figure 6).

While composites containing either lignin or glass fiber showed comparable track thicknesses (~700 μm), the formulation containing both GF and lignin (25DEALK_15GF) demonstrated larger tracks, with a thickness of approximately 1 mm. This increase can be attributed to the combined action of laser beam scattering induced by glass fibers and laser radiation absorption promoted by lignin. High magnifications revealed a hybrid microstructure incorporating features characteristic of both lignin-based and silica-based composites. Specifically, we observed branched carbonaceous structures in addition to molten glass spheres (Figure 8(c‴)).

**Figure 9:**
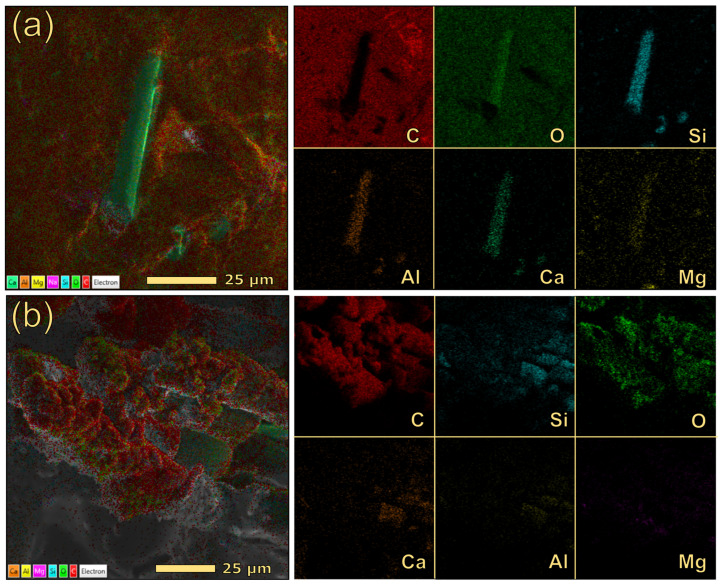
EDX elemental mapping for the 30GF sample. The images were recorded in the bulk region (**a**) and on a GF inside the conductive path (**b**). Individual maps are ordered by elemental abundance.

The morphological analysis of 30GF indicated the presence of exposed glass fibers, having lengths of 160–180 µm, in the region subjected to the laser process, in addition to a discrete number of molten glass spheres (Figure 8(b″)). To further elucidate the interaction between glass fibers, polymer, and laser radiation, EDX mapping was conducted on both the bulk material and the scribed path. Figure 9 presents layered composition maps (left) and individual elemental maps ordered by abundance (right). Analysis of the laser-irradiated region (Figure 9b) revealed that the glass fibers are entirely enveloped by carbonaceous material, resulting in a corresponding attenuation of the silicon signal intensity. Conversely, the analysis performed on an exposed glass fiber within a non-irradiated region (Figure 9a) demonstrated, as expected, that silicon is confined to the exposed fiber area, while carbon is primarily present in the surrounding polymeric matrix.

The previous results indicated that the incorporation of glass fibers into PBAT significantly modified the polymer’s response to laser scribing. While pristine PBAT primarily underwent ablation upon laser irradiation, GF-reinforced composites developed conductive tracks. Although PBAT itself forms only minimal char (3.7 wt%) during pyrolysis, silica-based fillers can stabilize the pyrolytic char of PBAT, which remains anchored to the silica structures, enabling electrical conductivity. In contrast, in the absence of thermally stable fillers, the carbonaceous residue fails to adhere to the substrate, resulting in predominant ablation of PBAT during laser treatments. These findings, observed in composites not containing a char-forming filler (e.g., lignin), demonstrated that glass fibers alone can effectively stabilize PBAT-derived carbonaceous residues.

**Figure 10:**
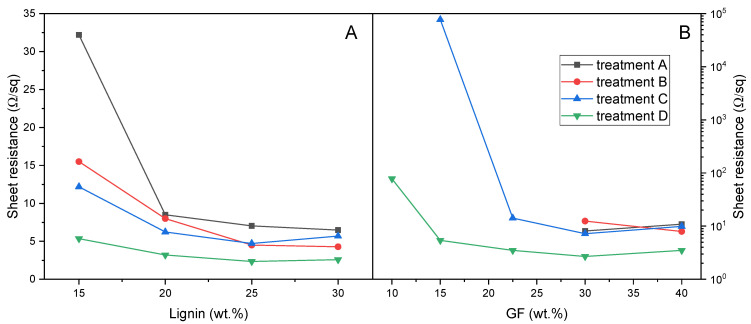
Comparison of the electrical properties in terms of sheet resistance of composites containing varying percentages of lignin (**A**) and GF (**B**) as a function of the laser treatment parameters.

Although a fully quantitative description of agglomeration and network density was not carried out here, the combined evidence from SEM/EDX, TGA-derived actual compositions and the smooth evolution of sheet resistance with lignin and GF contents (Figure 6, Figure 7 and Figure 10) supports the interpretation that, in the dealkaline lignin/GF systems, fillers are reasonably well dispersed at the micrometric scale and form extended conductive pathways rather than isolated agglomerates.

**Figure 11:**
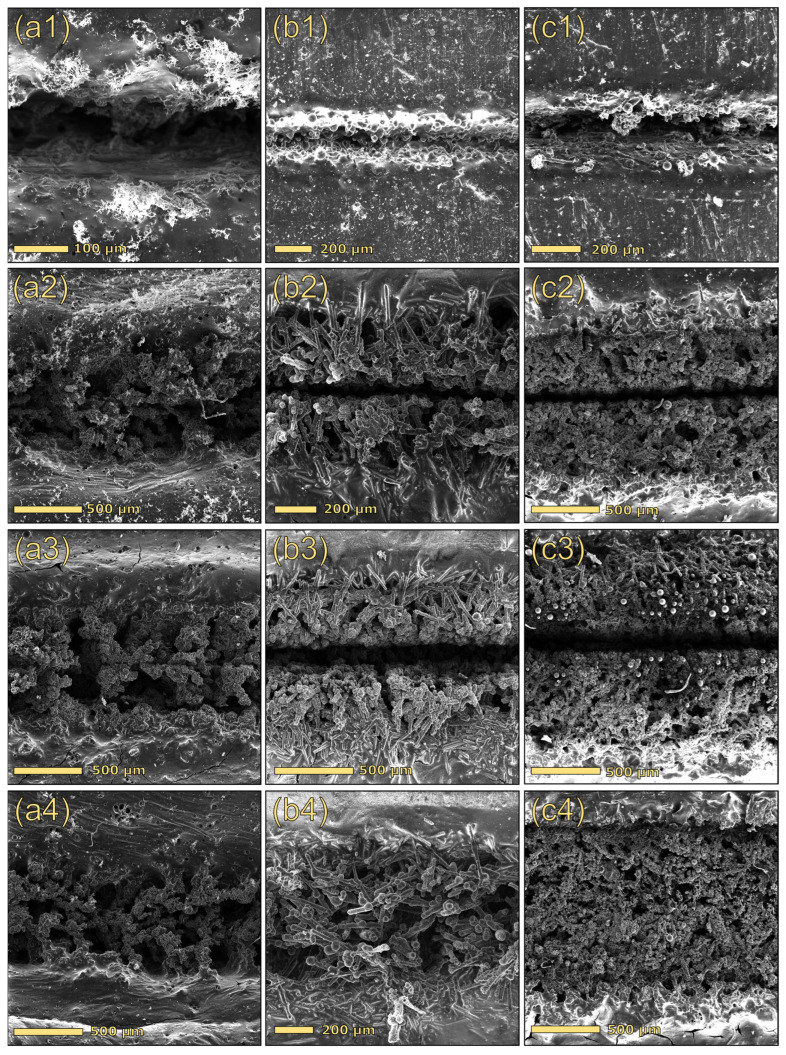
Top-view images of laser-scribed path on 15DEALK (**a1**–**a4**), 30GF (**b1**–**b4**), and 25DEALK_15GF (**c1**–**c4**). The tracks were obtained using only the pretreatment (1), treatment C (2), treatment D (3), and treatment B (4).

Motivated by these encouraging outcomes, we decided to further optimize the laser treatment by acting on additional processing parameters. Specifically, a pretreatment was introduced before the laser functionalization, employing elevated laser power (80%, 20 W) and speed (100 mm/s). Although this step did not yield a conductive track, it altered the local morphology by generating an ablated groove (Figure 11, row 1). The pre-ablated region was subsequently subjected to the laser process using the optimized parameters, i.e., speed 1 mm/s, power 8%, and surface-focused beam. This process, consisting of a pretreatment followed by laser functionalization, is designated as treatment C. Comparing the blue and black lines in Figure 10A, which correspond to the same laser process with (blue line) and without (black line) the pretreatment, it is evident that this initial step significantly reduced the sheet resistance. The pretreatment resulted in a significant improvement in electrical performance as well as enhanced track stability to mechanical stress.

The strong decrease in sheet resistance followed by a plateau at higher lignin/GF contents is indicative of percolation-like behavior, where a continuous conductive network is formed above a critical effective filler concentration. However, a quantitative percolation analysis (e.g., determination of the percolation threshold and critical exponent) would require an accurate estimation of the effective conductive fraction in the laser-modified regions and is therefore beyond the scope of this work.

Furthermore, the effect of repeated treatments on electrical conductivity was also examined by doubling the number of passes on the same track. Treatment D involved a pretreatment step (speed 100 mm/s and a power of 80%) followed by two consecutive laser treatments under optimized parameters (speed 1 mm/s, power 8%, surface-focused beam). As shown in Figure 10, the application of a pretreatment, followed by two consecutive treatments under optimal conditions (treatment D), produced a consistent and pronounced decrease in sheet resistance for both the lignin-based composites and those containing glass fibers (see the green line).

Interestingly, the lower sheet resistance reached ca. 1.7–2 Ω/sq with the presence of GF at 22–40 wt.%. These values are among the lowest reported for laser-engraved carbon tracks. For comparison, paper-based LIG (laser-induced graphene) electrodes typically show sheet resistances in the 14–30 Ω/sq range when used in disposable pH sensors or flexible microsupercapacitors [49]. LIG devices, exhibiting sheet resistance values in the range of 12–14 Ω/sq, have been effectively utilized as disposable electrochemical pH sensors, biosensors, and Hall effect sensors [44,50]. In comparison, laser-patterned polyurethane/graphene composites, offering superior conductivity as low as 2.5 Ω/sq, have been successfully implemented in applications including heaters, proximity sensors, and amperometric biosensors [51]. Recent reviews on bioderived LIG further indicate that lignocellulosic and lignin-based precursors typically yield sheet resistances in the 10–100 Ω/sq range, with best performing systems around ca. 10 Ω/sq [52].

In this context, the value of 1.7 Ω/sq achieved on our PBAT/lignin/GF substrate is fully comparable with, and in some cases superior to, state-of-the-art carbon conductors for flexible printed electronics.

The morphological analyses performed via electron microscopy (Figure 11) and optical microscopy (Figures S6–S8) confirmed that scribed path morphology varied significantly depending on both the treatment protocol and sample composition. Notably, track structure demonstrated overall improvement through surface pretreatment (reference tracks in Figure 8 vs. tracks in row 2 of Figure 11), whereas overfocus treatment (treatment B; Figure 11, row 4) yielded more homogeneous paths, lacking central grooves, with shallower penetration and greater surface exposure (refer to Figures S6–S8). Conversely, the increase in the number of sequential treatments promoted the formation of higher amounts of carbon within scribed channels, resulting in better-structured tracks.

Regarding compositional variations, microscopy analysis revealed a synergistic interaction between glass fibers and lignin. Specifically, tracks produced on composites containing both filler types (25DEALK_15GF; Figure 11, column c) exhibited enhanced morphological uniformity, improved mechanical stability, and frequently demonstrated lower sheet resistance values. The superior performance of hybrid composites is attributed to the complementary functions of the fillers. During CO_2_ laser irradiation, lignin acts as a carbon-forming precursor, generating conductive carbonaceous microstructures, whereas GF assists the process through multiple mechanisms. Specifically, GF scatters and redistributes laser energy, stabilizes the molten and partially carbonized regions, and physically anchors the nascent carbon network. This synergy results in hybrid carbon–GF channels that demonstrate reduced electrical resistance and superior mechanical robustness.

Our observations on PBAT/lignin/GF evidence the presence of a porous carbon network in the central region of the track, surrounded by zones of partial carbonization, and the strong dependence of morphology and conductivity on laser power, speed, and defocus are fully consistent with the deep thermal gradients and the localized overheating. These features can be rationalized with recent modelling and experimental studies on laser-induced graphene formation from lignin-rich precursors [48,53].

#### 3.2.2. Correlation of the Actual Filler Content with the Electrical Performance

Determining the actual composition was a crucial step in establishing the correlation between the electrical performance of the composites and their composition. To determine the actual content of PBAT, lignin, and silica, we formulated a three-variable system based on the assumption that the pyrolysis and ash residues generated from each composite correspond to the sum of the individual residues from PBAT, lignin, and silica, each weighted by its mass fraction in the material. The solution of the system is shown below.

$$x=\frac{num1}{\left(B-A\right)(CD-BD-CE+AE+BF-AF)}$$

$$y=\frac{num2}{\left(B-A\right)(CD-BD-CE+AE+BF-AF)}$$

$$z=\frac{BR_{800}-AR_{800}-BD+DR_{690}-ER_{690}+AE}{CD-BD-CE+AE+BF-AF}$$

The numerator of x and y is calculated with the following equations:

$$num1=A\left(FR_{690}-CR_{800}-ER_{690}+CE-BF+BR_{800}\right)+B(CR_{800}-FR_{690}-CE+ER_{690}+BF-BR_{800})$$

$$num2=A\left(BR_{800}+DR_{690}+CR_{800}-CD-FR_{690}-BF+AF-AR_{800}\right)+B(-DR_{690}+FR_{690}-CR_{800}+CD)$$

x, y, and z indicate the fraction of PBAT, lignin, and GF within the composite, respectively. R690 denotes the pyrolysis residue of the composite, while R800 corresponds to its ash. The residues of the pristine components are labeled in the following way:

- A and D indicate the pyrolysis and ash residues of PBAT, respectively.
- B and E indicate the pyrolysis and ash residues of lignin, respectively.
- C and F indicate the pyrolysis and ash residues of GF, respectively.

TGA was used to determine the pyrolysis residue and ash content. The pyrolysis residue was measured at 690 °C in a nitrogen environment, and the ash content was measured at 800 °C in an air environment.

First, TGA was carried out on pristine PBAT (Figure S2) and dealkaline lignin (Figure S1). Table 4 shows the average residue values calculated from at least three analyses performed for each component.

Lignin pyrolysis yielded 43.2% char, significantly higher than the char generated from PBAT (3.7%). Upon thermal oxidation, lignin produced a substantial ash residue (12.6%), primarily composed of O (38%), Na (30%), and S (29%). In contrast, PBAT yielded a minimal amount of ash, which mainly consisted of O (51%), Si (26%), and Mg (18%). The inorganic residue in lignin originates from its extraction process, whereas the ash from PBAT is likely attributable to process additives.

Since synergistic effects between components could impact the pyrolysis process and residue quantification, the system was first validated with six physical mixtures of known composition. These reference samples were prepared by precise gravimetric measurement of components in the ratios specified in Table 5 (first column), followed by mixing at ambient temperature. The results in Table 5 highlighted that the three-variable system yielded values in close agreement with the nominal compositions, with deviations under 1% (see values in brackets).

After confirming its validity, the system was used to determine the actual composition of the composites prepared via melt mixing. As shown in Table 6, substantial deviations were observed between the actual and nominal compositions for certain formulations, indicating inherent limitations in the compounding process. Furthermore, negative values were calculated for some components. However, considering the previous results on physical blends, these values (which did not exceed 1%) are within the experimental error and can therefore be considered zero.

**Figure 12:**
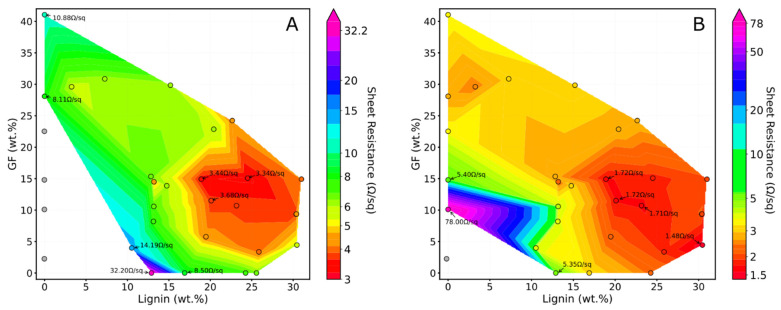
Color-coded maps correlating the actual concentrations of glass fibers (GF) and dealkaline lignin, calculated through thermogravimetric measurements, with the sheet resistance of the composites. (**A**) refers to treatment A, (**B**) corresponds to the optimized treatment, denoted as D. Experimental data points are represented as colored circles according to the measured sheet resistance values. Experimental points shown in grey correspond to traces whose resistance values were too high to be measured.

The results correlating the actual concentration of glass fibers and dealkaline lignin dispersed in the polymer matrix with the sheet resistance of the laser-scribed tracks are presented in Figure 12 as a color-coded map.

As shown in panel A, some of the composites containing only glass fibers, processed using the non-fully optimized parameters (treatment A), did not exhibit significant electrical properties, as their sheet resistance values exceeded the detection limit (>20 MΩ). However, following the optimization of the functionalization process using treatment D, it is evident that conductive tracks with measurable sheet resistance values can also be obtained for these samples. Overall, the optimized laser treatment substantially decreased the sheet resistance values of the composites. For instance, considering the formulation containing 28.1 wt% GF, the initial sheet resistance of 8.11 Ω/sq, obtained using the non-optimized parameters, decreases to 2.68 Ω/sq following process optimization. The overall color-coded map reveals the existence of an optimal range of lignin and GF concentrations that yields conductive tracks with markedly low sheet resistance. This range, regardless of the laser treatment, is between 10 wt% and 15 wt% for GF and between 20 wt% and 25 wt% for lignin. In the case of treatment A (non-optimized), the sheet resistance values within this concentration range are approximately 3.5 Ω/sq; in contrast, with the optimized parameters (treatment D), this value decreases to approximately 1.72 Ω/sq.

Although the laser functionalization in this study was performed on specimens with dimensions of a few centimeters, the CO_2_ engraving setup is capable of writing conductive paths with lengths up to several tens of centimeters. Furthermore, CO_2_ laser cutters with working areas of hundreds of centimeters, which are routinely employed in industrial practice, suggest the feasibility of scaling this process for large-area or highly patterned devices.

## 4. Conclusions

This study investigated the modification of electrical properties in polybutylene adipate terephthalate (PBAT)-based composites through CO_2_ laser treatment in an inert atmosphere. Through laser functionalization, the composite’s electrical behavior was locally modified, resulting in a transition from insulating to conductive behavior.

Initially, composites were prepared by incorporating different fillers (dealkaline lignin, alkaline lignin, hydrophilic nano silica, hydrophobic nano silica, and glass fibers) at fixed concentrations into the polymeric matrix. Among these, dealkaline lignin and glass fibers (GF) provided the optimal balance of electrical conductivity, mechanical properties, and processability. Subsequently, formulations with varying lignin (0–30 wt%) and GF (0–40 wt%) contents were prepared. These composites were subjected to laser treatment and characterized through electrical resistance measurements, morphological analyses via electron microscopy and optical microscopy, EDX, and TGA.

While laser irradiation of pure PBAT primarily caused polymer ablation, the inclusion of lignin or GF facilitated conductive path formation. SEM and EDX analyses revealed that the two fillers contributed differently to the conductive track formation. In PBAT/lignin composites, the interaction between the CO_2_ laser and lignin (a carbon precursor) produced conductive tracks with interconnected branched carbonaceous structures. In contrast, the laser treatment generated carbon-coated fibers alongside molten glass spheres in PBAT/GF composites. The high thermal stability of GF not only prevented PBAT ablation but also stabilized the limited carbonaceous residue derived from polymer pyrolysis. Additionally, the incorporation of GF altered the rheological properties of the polymer melt, resulting in sharply defined edges within the laser-modified regions.

The influence of filler concentration on electrical performance was also investigated. The results indicated an increase in electrical properties with filler loading up to a critical concentration, beyond which resistance plateaued. This trend was observed in both lignin- and GF-based composites.

To enhance the electrical performance, laser treatment parameters (speed, power, focal length, and number of repeated irradiations) were systematically optimized. The best electrical results were achieved using a protocol consisting of a pretreatment step (100 mm/s, 20 W) followed by two sequential treatments (1 mm/s, 2 W, focused beam). Although the pretreatment did not generate conductive tracks, it improved the mechanical stability of the final conductive paths. Conversely, increasing the number of sequential treatments enhanced carbon deposition and track uniformity.

This study demonstrated that hybrid composites containing both lignin and GF exhibited the best electrical properties. The lowest sheet resistance values were observed at GF concentrations between 10 and 15 wt% and lignin concentrations between 20 and 25 wt%. When the optimized laser treatment was performed on these composites, sheet resistance values of 1.7 Ω/sq were measured. The combined effect of lignin and GF led to the formation of branched carbonaceous structures and silica structures coated with carbon particles. The presence of this hybrid morphology led to improved track homogeneity and mechanical stability, in addition to high conductivity.

Future work will aim at quantitative temperature measurements during CO_2_ laser irradiation of PBAT/lignin/GF to more directly correlate local thermal history with the extent of carbonization and the resulting electrical properties. Furthermore, a statistical design of experiments, combining filler composition and laser parameters as factors and electrical/mechanical properties as responses, would allow quantitative design maps to be constructed.

Overall, this work establishes a viable route to tailor electrically conductive PBAT composites through controlled laser processing and filler selection.

## Figures and Tables

**Table 1:** Properties of the different types of lignin used in this study.

	pH	Methoxy Group (%) *	Ash (Sulfate) (%) *
Alkaline lignin	8.0–10.0	10.0–12.0	20.0–29.0
Dealkaline lignin	3.0–4.0	10.0–12.5	10.0–20.0

* Calculated on dry substance.

**Table 2:** Properties of the different types of silica used in this study.

Type of Silica	Properties
Hydrophilic silica	Fumed silica nanoparticles having a pH of 3.6–4.5.
Hydrophobic silica	Fumed silica nanoparticles having many trimethylsilyl groups in place of hydroxyl groups.
Glass fibers	Silica microfibers having a length of 160 µm and a nominal diameter of 13 µm.

**Table 3:** Tensile results of PBAT and its composites containing various types of lignin and silica.

	Young’s Modulus (MPa)	Yield Stress (MPa)	Stress at Break (MPa)	Nominal Strain at Break (%)
PBAT	119 ± 1	7.9 ± 0.2	10.9 ± 0.2	320 ± 20
30DEALK	292 ± 4	6.8 ± 0.1	6.2 ± 0.1	7.8 ± 0.7
30DEALK_5SI	207 ± 5	6.2 ± 0.2	5.9 ± 0.1	13 ± 4
30DEALK_5mSI	227 ± 9	6.1 ± 0.1	5.8 ± 0.2	12 ± 4
30DEALK_5GF	251 ± 6	6.2 ± 0.1	5.7 ± 0.1	8.5 ± 0.9
30ALK	260 ± 20	6.3 ± 0.1	5.7 ± 0.1	20 ± 3
30ALK_5SI	240 ± 20	6.5 ± 0.2	6.0 ± 0.2	18 ± 3
30ALK_5mSI	269 ± 2	6.9 ± 0.1	6.3 ± 0.1	13 ± 2
30ALK_5GF	250 ± 20	5.7 ± 0.2	5.3 ± 0.1	11 ± 4

**Table 4:** Average residues of PBAT, dealkaline lignin, and glass fibers expressed as weight percentages on the dry mass. PBAT and lignin residues were quantified by TGA.

	Pyrolysis Residue	Ash
PBAT	5.1 ± 0.2	1.4 ± 0.1
Dealkaline lignin	55.8 ± 0.9	12.6 ± 0.5
Glass fibers	100	100

**Table 5:** Percent composition of the physical mixtures calculated with the system of three variables. The values in brackets indicate the difference between the nominal and the actual percentage, the latter determined by gravimetry.

PBAT/DEALK/GF Theoretical Ratio	PBAT (%)	DEALK (%)	GF (%)
92.80/0/7.20	92.03 (−0.77)	0.77 (0.77)	7.20 (0)
59.89/0/40.11	60.43 (0.54)	−0.41 (−0.41)	39.98 (−0.13)
90.44/9.56/0	91.01 (0.57)	9.02 (−0.54)	−0.03 (−0.03)
58.68/41.32/0	58.53 (−0.15)	40.76 (−0.56)	0.71 (0.71)
0/44.63/55.37	0.13 (0.13)	44.27 (−0.36)	55.59 (0.22)
58.34/19.85/21.81	58.30 (−0.04)	20.04 (0.18)	21.67 (−0.14)

**Table 6:** Actual composition of the samples prepared by melt mixing, calculated via the system of three variables.

	PBAT (%)	DEALK (%)	GF (%)
PBAT_5GF	97.5	0.1	2.4
PBAT_10GF	90.7	−0.8	10.1
PBAT_15GF	86.0	−0.8	14.8
PBAT_22.5GF	77.9	−0.4	22.5
PBAT_30GF	72.3	−0.4	28.1
PBAT_40GF	59.7	−0.8	41.1
PBAT_5DEALK_30GF	67.1	3.3	29.6
PBAT_10DEALK_30GF	61.8	7.3	30.9
PBAT_15DEALK	86.9	12.9	0.2
PBAT_15DEALK_5GF	85.5	10.5	4.0
PBAT_15DEALK_7.5GF	78.7	13.1	8.2
PBAT_15DEALK_10GF	76.2	13.2	10.6
PBAT_15DEALK_15GF	72.3	13.2	14.5
PBAT_15DEALK_30GF	54.9	15.2	29.9
PBAT_20DEALK	82.9	16.9	0.2
PBAT_20DEALK_5GF	74.7	19.5	5.8
PBAT_20DEALK_10GF	68.4	20.1	11.5
PBAT_20DEALK_15GF	66.2	18.9	14.9
PBAT_20DEALK_25GF	53.1	22.7	24.2
PBAT_25DEALK	75.8	24.3	−0.1
PBAT_25DEALK_5GF	70.7	25.9	3.4
PBAT_25DEALK_10GF	66.1	23.2	10.7
PBAT_25DEALK_15GF	59.4	24.5	15.1
PBAT_30DEALK	75.1	24.3	0.6
PBAT_30DEALK_5GF	65.1	30.5	4.4
PBAT_30DEALK_10GF	60.1	30.4	9.4
PBAT_30DEALK_15GF	54.1	31.0	14.9

## Data Availability

The original contributions presented in the study are included in the article/Supplementary Material; further inquiries can be directed to the corresponding author.

## Supplementary Materials

The following supporting information can be downloaded at: https://www.mdpi.com/article/10.3390/polym17233144/s1. Table S1: List of the composites prepared in this study and their theoretical composition; Figure S1: TGA of dealkaline lignin. The first part of the curve (from room temperature to 150 °C) corresponds to the moisture removal. The dashed line at 700 °C represents the transition of the gas from nitrogen to air; Figure S2: TGA of PBAT. The dashed line at 700 °C represents the transition of the gas from nitrogen to air; Table S2: Electrical sheet resistance values of PBAT-based composites subjected to different treatments with a CO2 laser. The values in the table are expressed as Ω/sq; Figure S3: Stress–strain curves of PBAT-based composites; Figure S4: Image of PBAT after laser treatments A (on the left) and C (on the right); Figure S5: EDX elemental maps of a laser-treated region on 30DEALK_5GF. Individual maps are ordered by elemental abundance; Figure S6: Optical micrographs of conductive tracks fabricated via laser scribing on 25DEALK_15GF. Panels (a), (b), (c), and (d) show top-view images of tracks produced by laser treatments A, B, C, and D, respectively. The corresponding cross-sectional profiles are displayed in panels (a′), (b′), (c′), and (d′); Figure S7: Optical micrographs of conductive tracks fabricated via laser scribing on 30GF. Panels (a), (b), (c), and (d) show top-view images of tracks produced by laser treatments A, B, C, and D, respectively. The corresponding cross-sectional profiles are displayed in panels (a′), (b′), (c′), and (d′); Figure S8: Optical micrographs of conductive tracks fabricated via laser scribing on 15DEALK. Panels (a), (b), (c), and (d) show top-view images of tracks produced by laser treatments A, B, C, and D, respectively. The corresponding cross-sectional profiles are displayed in panels (a′), (b′), (c′), and (d′).

## References

[1] United Nations Institute for Training and Research Global E-Waste Monitor 2024: Electronic Waste Rising Five Times Faster than Documented E-Waste Recycling. https://unitar.org/about/news-stories/press/global-e-waste-monitor-2024-electronic-waste-rising-five-times-faster-documented-e-waste-recycling.

[2] Tan M.J., Owh C., Chee P.L., Kyaw A.K.K., Kai D., Loh X.J. (2016). Biodegradable Electronics: Cornerstone for Sustainable Electronics and Transient Applications. J. Mater. Chem. C Mater..

[3] Cao Y., Uhrich K.E. (2019). Biodegradable and Biocompatible Polymers for Electronic Applications: A Review. J. Bioact. Compat. Polym..

[4] Shim J.-S., Rogers J.A., Kang S.-K. (2021). Physically Transient Electronic Materials and Devices. Mater. Sci. Eng. R Rep..

[5] Li R., Wang L., Kong D., Yin L. (2018). Recent Progress on Biodegradable Materials and Transient Electronics. Bioact. Mater..

[6] Peng X., Dong K., Wu Z., Wang J., Wang Z.L. (2021). A Review on Emerging Biodegradable Polymers for Environmentally Benign Transient Electronic Skins. J. Mater. Sci..

[7] Liu H., Jian R., Chen H., Tian X., Sun C., Zhu J., Yang Z., Sun J., Wang C. (2019). Application of Biodegradable and Biocompatible Nanocomposites in Electronics: Current Status and Future Directions. Nanomaterials.

[8] Jamshidi R., Taghavimehr M., Chen Y., Hashemi N., Montazami R. (2022). Transient Electronics as Sustainable Systems: From Fundamentals to Applications. Adv. Sustain. Syst..

[9] Song Z., Liu Z., Zhao L., Chang C., An W., Zheng H., Yu S. (2022). Biodegradable and Flexible Capacitive Pressure Sensor for Electronic Skins. Org. Electron..

[10] Zhu B., Wang H., Leow W.R., Cai Y., Loh X.J., Han M., Chen X. (2016). Silk Fibroin for Flexible Electronic Devices. Adv. Mater..

[11] Zhou Z., Zhang H., Liu J., Huang W. (2021). Flexible Electronics from Intrinsically Soft Materials. Giant.

[12] Brunella V., Rossatto B.G., Scarano D., Cesano F. (2021). Thermal, Morphological, Electrical Properties and Touch-Sensor Application of Conductive Carbon Black-Filled Polyamide Composites. Nanomaterials.

[13] Cesano F., Rattalino I., Demarchi D., Bardelli F., Sanginario A., Gianturco A., Veca A., Viazzi C., Castelli P., Scarano D. (2013). Structure and Properties of Metal-Free Conductive Tracks on Polyethylene/Multiwalled Carbon Nanotube Composites as Obtained by Laser Stimulated Percolation. Carbon.

[14] Mastropasqua C., Veca A., Damin A., Brunella V., Cesano F. (2022). Functional Piezoresistive Polymer Composites Based on CO_2_ Laser-Irradiated Graphene Oxide-Loaded Polyurethane: Morphology, Structure, Electrical and Piezoresistive Properties. Nanomaterials.

[15] Zhang H., Yan Q., Peng Y., Cai Z., Wan C. (2024). Upgrading Lignin into Graphene-Based Materials: State of the Art and Perspectives. Adv. Energy Sustain. Res..

[16] Cesano F., Uddin M.J., Damin A., Scarano D. (2021). Multifunctional Conductive Paths Obtained by Laser Processing of Non-Conductive Carbon Nanotube/Polypropylene Composites. Nanomaterials.

[17] Wang M., Yang Y., Gao W. (2021). Laser-Engraved Graphene for Flexible and Wearable Electronics. Trends Chem..

[18] Sarotto E., Brunella V., Cesano F. (2024). Graphene-Loaded Recycled PET: Exploring and Enhancing Electrical Conductivity through Processing and Laser Treatments near the Electrical Percolation Threshold. Sustain. Mater. Technol..

[19] Aversa C., Barletta M., Cappiello G., Gisario A. (2022). Compatibilization Strategies and Analysis of Morphological Features of Poly(Butylene Adipate-Co-Terephthalate) (PBAT)/Poly(Lactic Acid) PLA Blends: A State-of-Art Review. Eur. Polym. J..

[20] Itabana B.E., Mohanty A.K., Dick P., Sain M., Bali A., Tiessen M., Lim L., Misra M. (2024). Poly (Butylene Adipate-Co-Terephthalate) (PBAT)—Based Biocomposites: A Comprehensive Review. Macromol. Mater. Eng..

[21] Kim K.S., Yoo J., Shim J.S., Ryu Y.I., Choi S., Lee J.Y., Lee H.M., Koo J., Kang S.K. (2022). Biodegradable Molybdenum/Polybutylene Adipate Terephthalate Conductive Paste for Flexible and Stretchable Transient Electronics. Adv. Mater. Technol..

[22] Maciel C.C., de Barros A., Mazali I.O., Ferreira M. (2023). Flexible Biodegradable Electrochemical Sensor of PBAT and CNDs Composite for the Detection of Emerging Pollutants. J. Electroanal. Chem..

[23] Wang H.-M., Wang B., Yuan T.-Q., Zheng L., Shi Q., Wang S.-F., Song G.-Y., Sun R.-C. (2020). Tunable, UV-Shielding and Biodegradable Composites Based on Well-Characterized Lignins and Poly(Butylene Adipate-co-Terephthalate). Green Chem..

[24] Farsi M. (2012). Thermoplastic Matrix Reinforced with Natural Fibers: A Study on Interfacial Behavior. Some Critical Issues for Injection Molding.

[25] Popescu C.M. (2017). Wood as Bio-Based Building Material. Performance of Bio-Based Building Materials.

[26] Wang H.-M., Yuan T.-Q., Song G.-Y., Sun R.-C. (2021). Advanced and Versatile Lignin-Derived Biodegradable Composite Film Materials toward a Sustainable World. Green Chem..

[27] Leng E., Guo Y., Chen J., Liu S., Jiaqiang E., Xue Y. (2022). A Comprehensive Review on Lignin Pyrolysis: Mechanism, Modeling and the Effects of Inherent Metals in Biomass. Fuel.

[28] Li Y., Chen M., Shi Q.-S., Xie X., Guo Y. (2024). Biomass Fractionation Techniques Impact on the Structure and Antioxidant Properties of Isolated Lignins. Sep. Purif. Technol..

[29] Zhang M., Tian R., Tang S., Wu K., Wang B., Liu Y., Zhu Y., Lu H., Liang B. (2023). The Structure and Properties of Lignin Isolated from Various Lignocellulosic Biomass by Different Treatment Processes. Int. J. Biol. Macromol..

[30] Wang S., Ru B., Lin H., Sun W., Luo Z. (2015). Pyrolysis Behaviors of Four Lignin Polymers Isolated from the Same Pine Wood. Bioresour. Technol..

[31] Chen W., Tao X., Shi X., Guo W., Wang Y., Liu B., Yang H. (2024). Insight into Catalytic Effects of Alkali Metal Salts Addition on Bamboo and Cellulose Pyrolysis. npj Mater. Sustain..

[32] Gu X., Hou J., Ai S. (2022). Effect of Silane Modified Nano-SiO_2_ on the Mechanical Properties and Compatibility of PBAT/Lignin Composite Films. J. Appl. Polym. Sci..

[33] Xiong S.-J., Pang B., Zhou S.-J., Li M.-K., Yang S., Wang Y.-Y., Shi Q., Wang S.-F., Yuan T.-Q., Sun R.-C. (2020). Economically Competitive Biodegradable PBAT/Lignin Composites: Effect of Lignin Methylation and Compatibilizer. ACS Sustain. Chem. Eng..

[34] Yang X., Zhong S. (2020). Properties of Maleic Anhydride-modified Lignin Nanoparticles/Polybutylene Adipate-co-terephthalate Composites. J. Appl. Polym. Sci..

[35] Liu Y., Liu S., Liu Z., Lei Y., Jiang S., Zhang K., Yan W., Qin J., He M., Qin S. (2021). Enhanced Mechanical and Biodegradable Properties of PBAT/Lignin Composites via Silane Grafting and Reactive Extrusion. Compos. B Eng..

[36] Mai V.-D., Kwon G., Jang Y., Min J., Han J., Kim S.-K. (2024). High-Performance and Economic Biodegradable Composites Based on Polybutylene Adipate Terephthalate and Modified Lignin. Polym. Test..

[37] Kim J., Bang J., Park S., Jung M., Jung S., Yun H., Kim J.-H., Choi I.-G., Kwak H.W. (2023). Enhanced Barrier Properties of Biodegradable PBAT/Acetylated Lignin Films. Sustain. Mater. Technol..

[38] Xu H., Shaoyu J., Jin J., Li M., Ji L., Zhuang W., Tang C., Chang Z., Ying H., Zhu C. (2024). Fabrication of PBAT/Lignin Composite Foam Materials with Excellent Foaming Performance and Mechanical Properties via Grafting Esterification and Twin-Screw Melting Free Radical Polymerization. Collagen Leather.

[39] Botta L., Titone V., Teresi R., Scarlata M.C., Lo Re G., La Mantia F.P., Lopresti F. (2022). Biocomposite PBAT/Lignin Blown Films with Enhanced Photo-Stability. Int. J. Biol. Macromol..

[40] Kargarzadeh H., Kobylińska A., Antos-Bielska M., Krzyżowska M., Gałęski A. (2024). Exploring the Potential of Lignin Nanoparticles in Enhancing the Mechanical, Thermal, and Bioactive Properties of Poly (Butylene Adipate-Co-Terephthalate). Int. J. Biol. Macromol..

[41] Fang W., Yang S., Wang X.-L., Yuan T.-Q., Sun R.-C. (2017). Manufacture and Application of Lignin-Based Carbon Fibers (LCFs) and Lignin-Based Carbon Nanofibers (LCNFs). Green Chem..

[42] Zhang W., Yin J., Wang C., Zhao L., Jian W., Lu K., Lin H., Qiu X., Alshareef H.N. (2021). Lignin Derived Porous Carbons: Synthesis Methods and Supercapacitor Applications. Small Methods.

[43] Wen A., Wang C., Nong J., Hu C. (2025). Rapid Construction of Robust and Hydrophilic Lignin-Derived Laser-Induced Graphene Electrodes Using PVA-Free Dopants. Carbon.

[44] Funayama R., Hayashi S., Terakawa M. (2023). Laser-Induced Graphitization of Lignin/PLLA Composite Sheets for Biodegradable Triboelectric Nanogenerators. ACS Sustain. Chem. Eng..

[45] Lei Y., Alshareef A.H., Zhao W., Inal S. (2020). Laser-Scribed Graphene Electrodes Derived from Lignin for Biochemical Sensing. ACS Appl. Nano Mater..

[46] Zhang H., Li Q., Hammond K.D., He X., Lin J., Wan C. (2024). Probing Laser-Induced Structural Transformation of Lignin into Few-Layer Graphene. Green Chem..

[47] Mallick P.K. (2018). 2.18 Particulate Filled and Short Fiber Reinforced Polymer Composites. Comprehensive Composite Materials II.

[48] Ghavipanjeh A., Sadeghzadeh S. (2024). Simulation and Experimental Evaluation of Laser-Induced Graphene on the Cellulose and Lignin Substrates. Sci. Rep..

[49] Pinheiro T., Rosa A., Ornelas C., Coelho J., Fortunato E., Marques A.C., Martins R. (2023). Influence of CO_2_ Laser Beam Modelling on Electronic and Electrochemical Properties of Paper-Based Laser-Induced Graphene for Disposable PH Electrochemical Sensors. Carbon. Trends.

[50] Mao X., Wang Y., Li H., Zhao P., Zhao N., Xie B., Zhou Y. (2025). Laser-Induced Graphene Based on the Controllable Angle between Two Irradiation Steps for the Fabrication of Flexible Sensor. J. Mater. Chem. C Mater..

[51] Parkula V., Licata A., Valorosi F., Kovtun A., Scidà A., Ruani G., Liscio F., Zanardi C., Candini A., Bertocchi F. (2024). Laser-Patterned Polyurethane Composites with Graphene, Graphene Oxide, and Aramid Fibers for the Production of Electric Circuits, Sensors, and Heaters. ACS Appl. Nano Mater..

[52] Bressi A.C., Dallinger A., Steksova Y., Greco F. (2023). Bioderived Laser-Induced Graphene for Sensors and Supercapacitors. ACS Appl. Mater. Interfaces.

[53] Mahmood F., Zhang H., Lin J., Wan C. (2020). Laser-Induced Graphene Derived from Kraft Lignin for Flexible Supercapacitors. ACS Omega.

