# Supplementary material for: From Waste to Wires: PBAT/Lignin Biocomposites Functionalized by a CO2 Laser for Transient Electronics
Source: Polymers (Basel). 2025 Nov 26;17(23):3144. doi: 10.3390/polym17233144 (PMC12693890; doi:10.3390/polym17233144)
Supplement: Supplementary file 1 [file polymers-17-03144-s001.zip › polymers-3989956-supplementary.pdf]

Supplementary information

Table S1: List of the composites prepared in this study and their theoretical composition.

Sample name	PBAT (%)	ALK * (%)	DEALK * (%)	SI * (%)	mSI * (%)	GF * (%)
PBAT	100	–	–	–	–	–
30ALK	70	30	–	–	–	–
30ALK_5SI	65	30	–	5	–	–
30ALK_5mSI	65	30	–	–	5	–
30ALK_5GF	65	30	–	–	–	5
30DEALK	70	–	30	–	–	–
30DEALK_5SI	65	–	30	5	–	–
30DEALK_5mSI	65	–	30	–	5	–
30DEALK_5GF	65	–	30	–	–	5
5GF	95	–	–	–	–	5
10GF	90	–	–	–	–	10
15GF	85	–	–	–	–	15
22.5GF	77.5	–	–	–	–	22.5
30GF	70	–	–	–	–	30
40GF	60	–	–	–	–	40
5DEALK_30GF	65	–	5	–	–	30
10DEALK_30GF	60	–	10	–	–	30
15DEALK	85	–	15	–	–	–
15DEALK_5GF	80	–	15	–	–	5
15DEALK_7.5GF	77.5	–	15	–	–	7.5
15DEALK_10GF	75	–	15	–	–	10
15DEALK_15GF	70	–	15	–	–	15
15DEALK_30GF	55	–	15	–	–	30
20DEALK	80	–	20	–	–	–
20DEALK_5GF	75	–	20	–	–	5
20DEALK_10GF	70	–	20	–	–	10
20DEALK_15GF	65	–	20	–	–	15
20DEALK_25GF	55	–	20	–	–	25
25DEALK	75	–	25	–	–	–
25DEALK_5GF	70	–	25	–	–	5
25DEALK_10GF	65	–	25	–	–	10
25DEALK_15GF	60	–	25	–	–	15
30DEALK_10GF	60	–	30	–	–	10
30DEALK_15GF	55	–	30	–	–	15

* DEALK: dealkaline lignin; ALK: alkaline lignin; SI: hydrophilic nano silica; mSI: hydrophobic nano silica; GF: glass fibers.

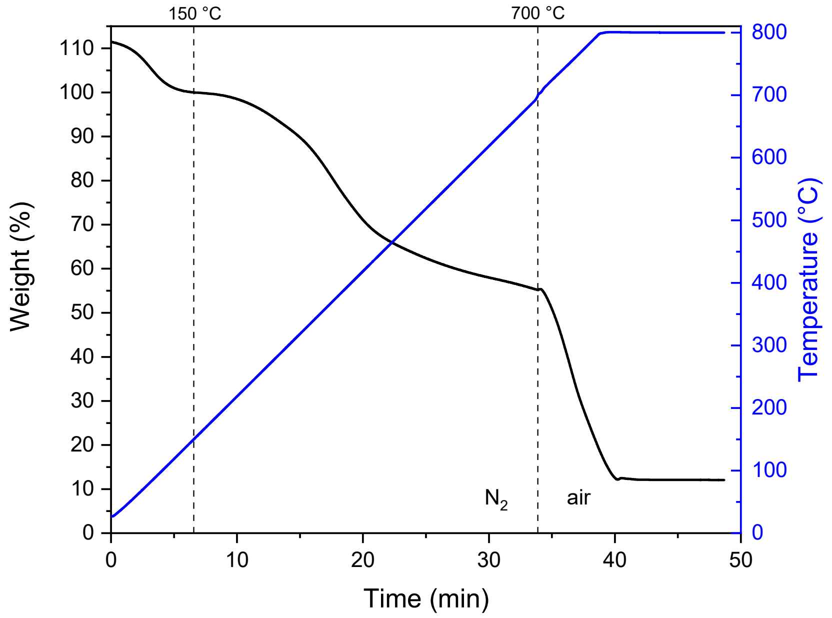

Figure S1: TGA of dealkaline lignin. The first part of the curve (from room temperature to 150 °C) corresponds to the moisture removal. The dashed line at 700 °C represents the transition of the gas from nitrogen to air.

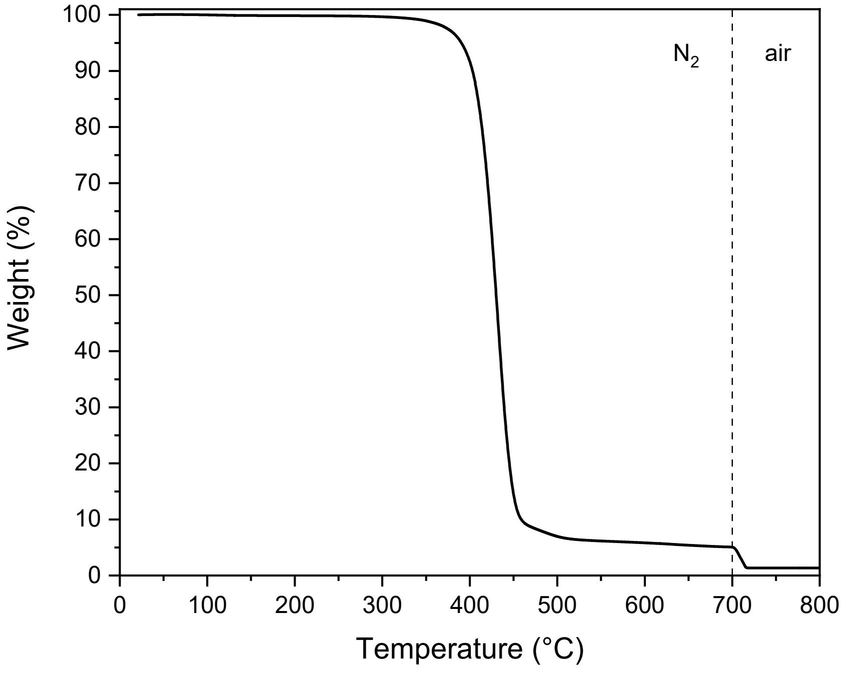

Figure S2: TGA of PBAT. The dashed line at 700 °C represents the transition of the gas from nitrogen to air.

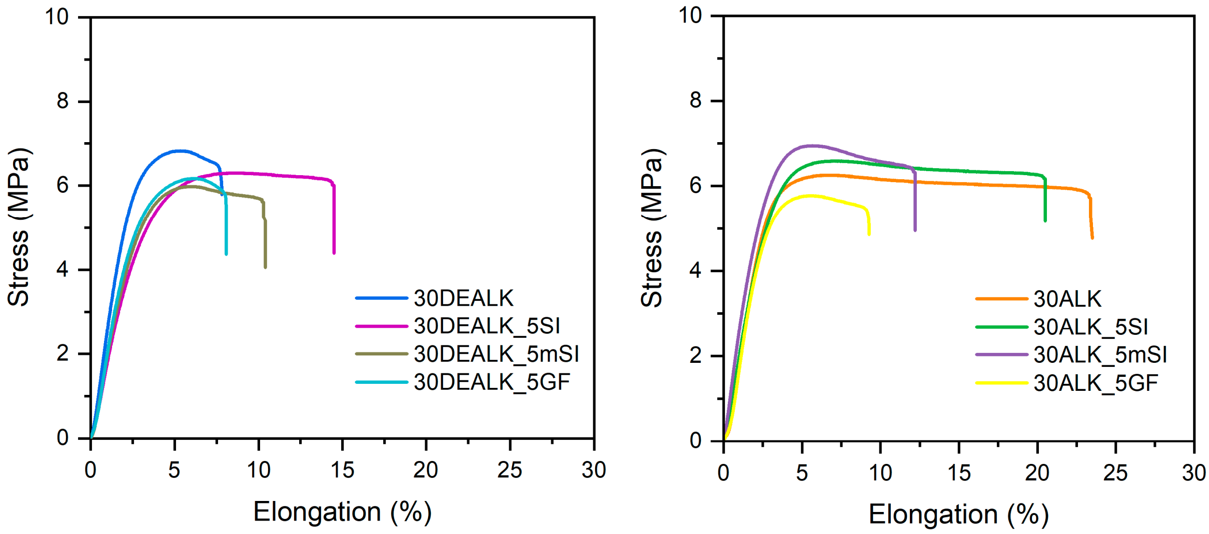

Figure S3: Stress-strain curves of PBAT-based composites.

Table S2: Electrical sheet resistance values of PBAT-based composites subjected to different treatments with a CO_2 laser. The values in the table are expressed as Ω/sq.

	Treatment A	Treatment B
PBAT	n.a.	n.a.
30DEALK	7.18	4.92
30ALK	60	260
30DEALK_5SI	3.01	3.56
30ALK_5SI	2.61	3.34
30DEALK_5mSI	4.63	3.35
30ALK_5mSI	5.95	5.15
30DEALK_5GF	4.41	3.86
30ALK_5GF	5.49	6.51

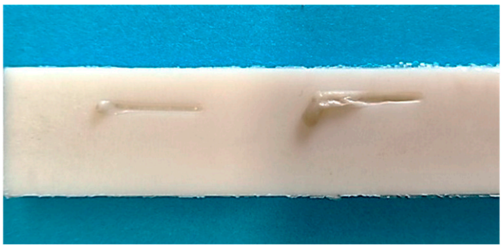

Figure S4: Image of PBAT after laser treatments A (on the left) and C (on the right).

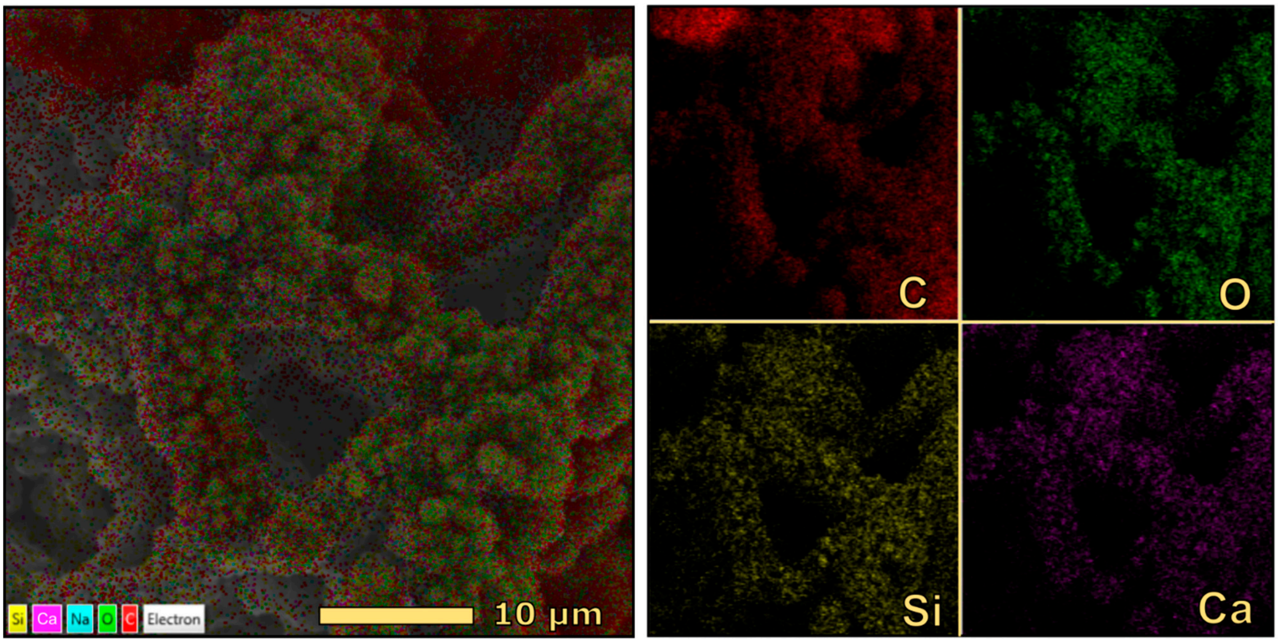

Figure S5: EDX elemental maps of a laser-treated region on 30DEALK_5GF. Individual maps are ordered by elemental abundance.

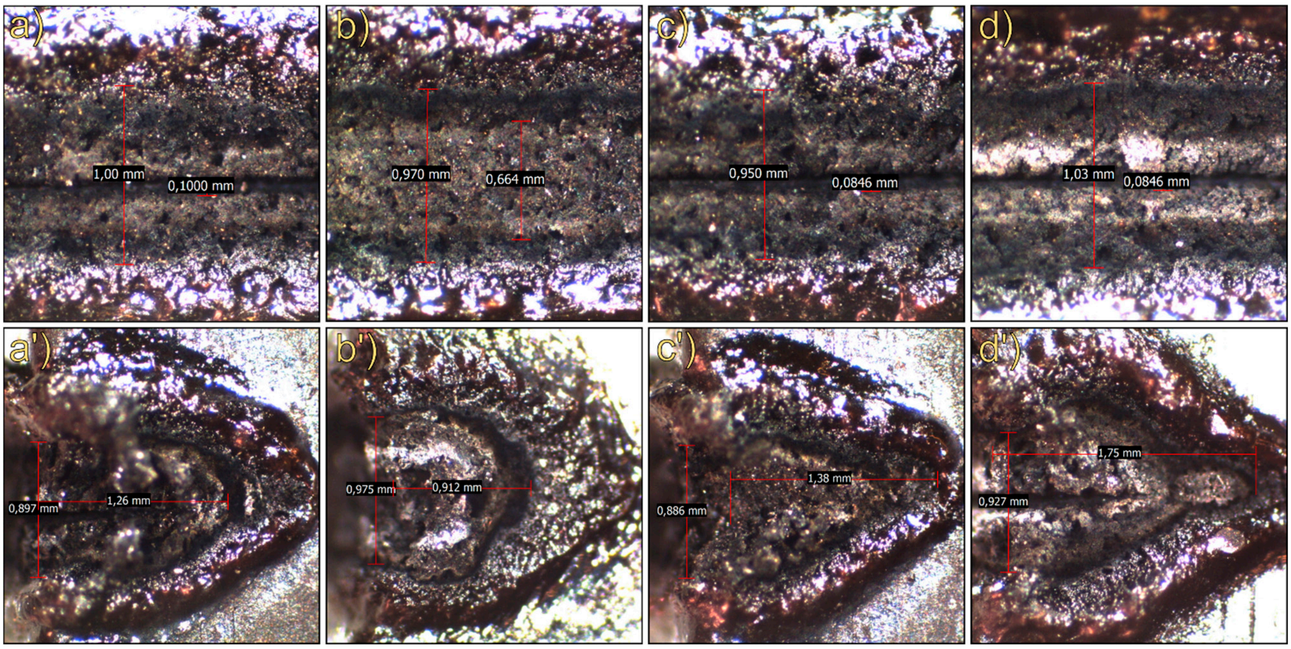

Figure S6: Optical micrographs of conductive tracks fabricated via laser scribing on 25DEALK_15GF. Panels (a), (b), (c), and (d) show top-view images of tracks produced by laser treatments A, B, C, and D, respectively. The corresponding cross-sectional profiles are displayed in panels (a′), (b′), (c′), and (d′)

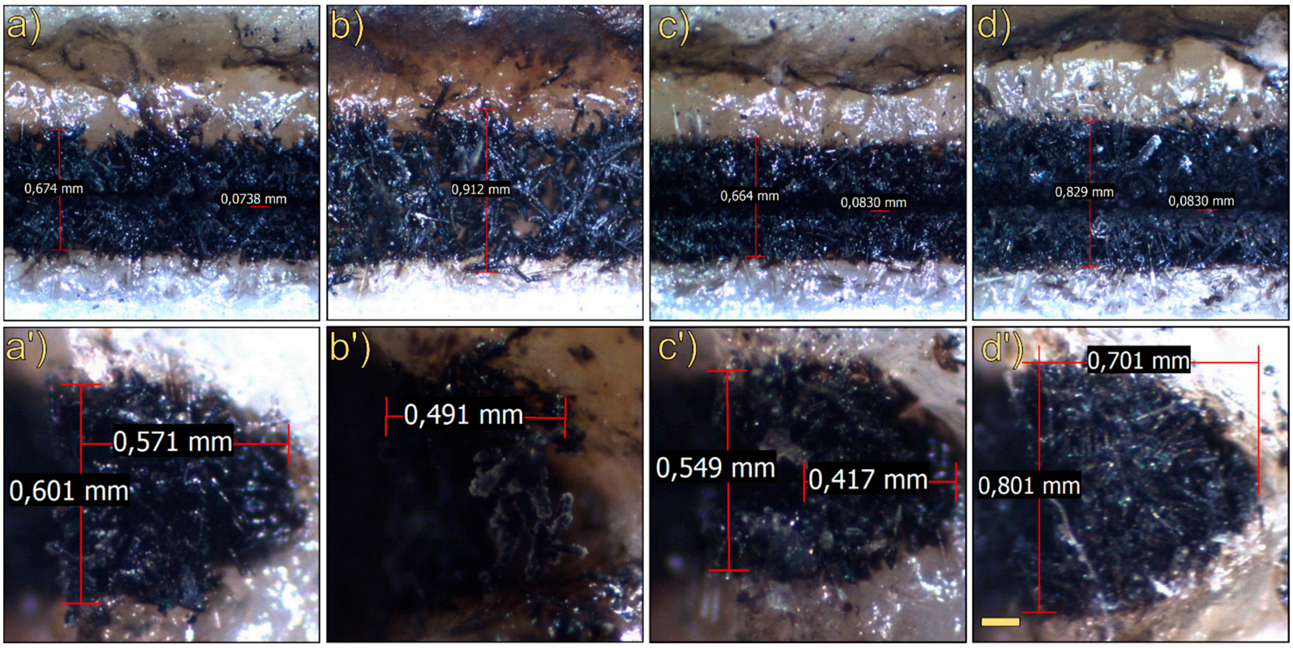

Figure S7: Optical micrographs of conductive tracks fabricated via laser scribing on 30GF. Panels (a), (b), (c), and (d) show top-view images of tracks produced by laser treatments A, B, C, and D, respectively. The corresponding cross-sectional profiles are displayed in panels (a′), (b′), (c′), and (d′).

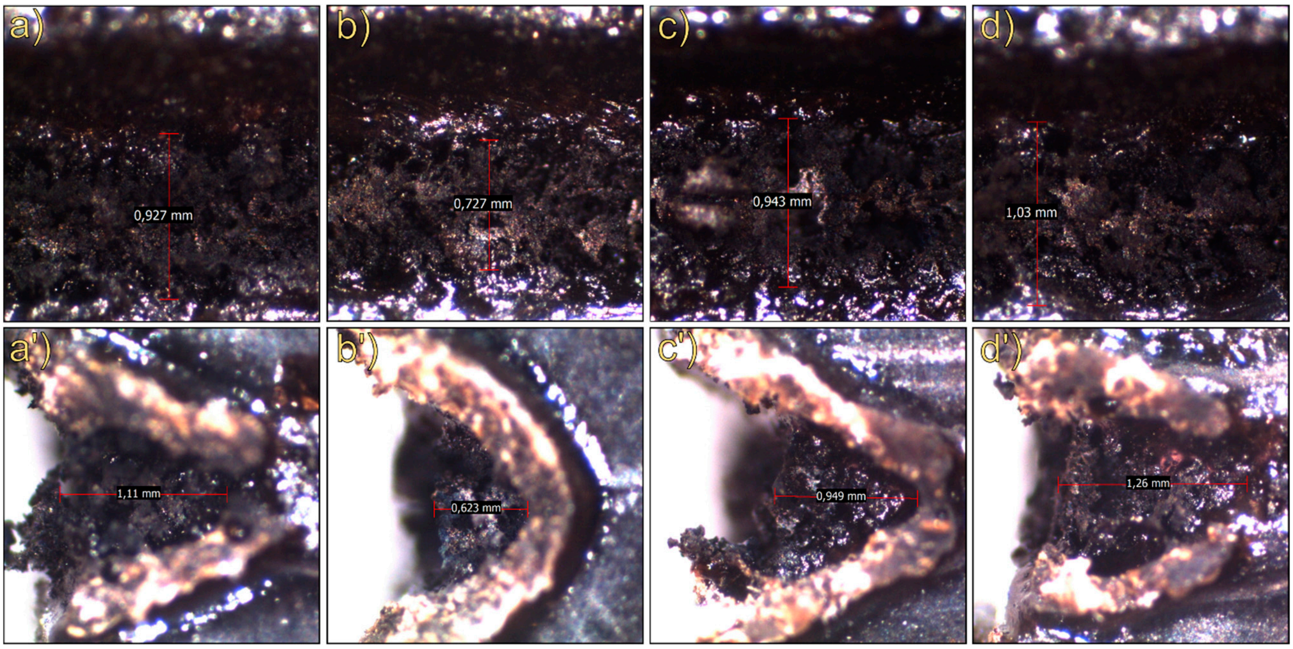

Figure S8: Optical micrographs of conductive tracks fabricated via laser scribing on 15DEALK. Panels (a), (b), (c), and (d) show top-view images of tracks produced by laser treatments A, B, C, and D, respectively. The corresponding cross-sectional profiles are displayed in panels (a′), (b′), (c′), and (d′).
